# 

**Published:** 1874-06

**Authors:** 


**TERMS, ALWAYS IN ADVANCE:** One copy, per annum.... $3 00

☞ Yearly subscriptions must begin with the January number.

☞ Persons who send subscriptions for the JOURNAL will please consider the first number received after subscription as a sufficient receipt for money sent.

☞ We cannot return rejected manuscripts.

